# Regional Cerebral Blood-Flow with 99mTc-ECD Brain Perfusion SPECT in Landau-Kleffner Syndrome: Report of Two Cases

**DOI:** 10.1155/2014/617343

**Published:** 2014-04-29

**Authors:** Reza Nemati, Iraj Nabipour, Hamid Javadi, Negar Chabi, Majid Assadi

**Affiliations:** ^1^Department of Neurology, Bushehr Medical Center Hospital, Bushehr University of Medical Sciences, Bushehr 7533934698, Iran; ^2^The Persian Gulf Tropical and Infectious Diseases Research Centre, Bushehr University of Medical Sciences, Bushehr 7533934698, Iran; ^3^Golestan Research Center of Gastroenterology and Hepatology (GRCGH), Golestan University of Medical Sciences (GUOMS), Gorgan 4917765181, Iran; ^4^The Persian Gulf Nuclear Medicine Research Center, Bushehr University of Medical Sciences, Bushehr 7533934698, Iran

## Abstract

Landau-Kleffner syndrome (LKS) is a rare childhood disorder characterized by acquired aphasia and epilepsy. 99mTc-ECD SPECT imaging was performed in two right-handed children with LKS. A relative decrease in perfusion was found in the left frontal-temporal cortices of both patients as well as in the left and right parietal cortices of one patient with aphasia, without clinical epilepsy. The degree of regional cerebral perfusion impairment did not correlate with the severity of the clinical and EEG abnormalities, but the area of hypoperfusion was compatible with the speech area of the brain. Overall, although asymmetrical temporoparietal perfusion appears as a common finding in LKS, SPECT findings in LKS alone cannot elucidate the pathogenic features of the disorder in the brain. Here, we present two cases of LKS in which we investigated SPECT perfusion scans.

## 1. Introduction

Landau-Kleffner syndrome (LKS) is considered to be an acquired epileptic aphasia that affects 0.2% of children with epilepsy [1]. The main clinical features of Landau-Kleffner syndrome consist of acquired childhood aphasia, paroxysmal EEG abnormalities, absence of focal brain lesions, and spontaneous stabilization after a variable length of time [2]. The pathophysiology of this syndrome is not fully understood. The neuroimaging studies using CT scans and MRIs, which are occasionally used as diagnostic criteria in these patients, are usually normal [2]. However, temporal lobe abnormalities are often observed in brain perfusion and glucose metabolism examinations as functional modalities using SPECT and PET on LKS patients [2, 3].

Here, we present two cases of LKS and the investigated perfusion scans of those two patients using SPECT.

## 2. Case Report

### 2.1. Case  1

**Figure 1:**
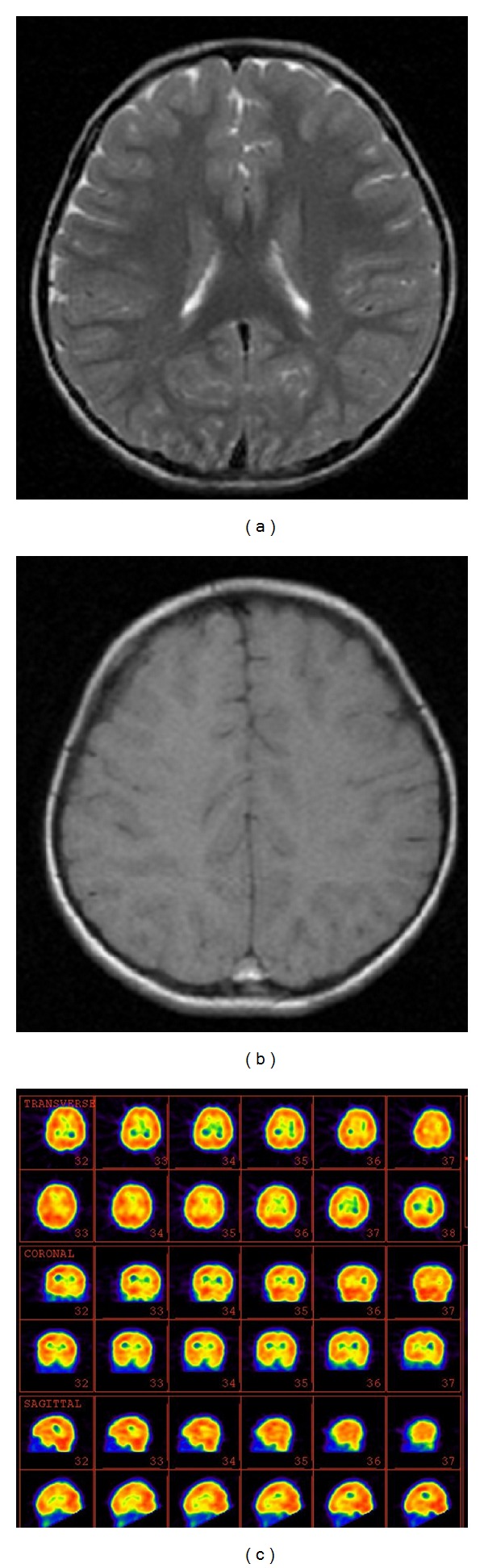
((a), (b)) The MRI images of a 6-year-old boy with Landau-Kleffner syndrome (LKS). (c) 99mTc-ECD brain perfusion SPECT image showed hypoperfusion in the left inferior frontal, left temporal, and also left thalamic regions. The upper rows indicate transverse 99mTc-ECD SPECT images. The middle rows indicate coronal 99mTc-ECD SPECT images. The lower rows indicate sagittal 99mTc-ECD SPECT images.

A six-year-old right-handed male presented with an acute onset of seizures during the first year of his life. His epilepsy was refractory to polytherapy, and the family history was negative for hearing difficulties, communication disorders, seizures, or other neurological disorders. His neurological examinations between seizures were normal, and there was no evidence of encephalitis. Additionally, there was no evidence of a neurometabolic disorder upon extensive investigation. The patient's blood glucose and lactate levels were normal, as were the CT head scan and subsequent MRI scan (Figures 1(a) and 1(b)). The EEG showed bilateral generalized high voltage slow activity, interrupted with sharp and slow activity which was more prominent over the left hemisphere. He was treated with a combination of anticonvulsants, including primidone, sodium valproate, lamotrigine, carbamazepine, levitiracetam, and prednisolone, but he had intractable seizures. In addition to refractory epilepsy, the patient developed a receptive and expressive language disorder in year 4 that deteriorated during one follow-up.

This patient's hearing was normal, and in year 6 he exhibited refractory epilepsy and global aphasia. The interictal brain perfusion SPECT in sleep state showed decreased perfusion in the left inferior frontal, left temporal, and left thalamic regions (Figure 1(c)).

### 2.2. Case  2

**Figure 2:**
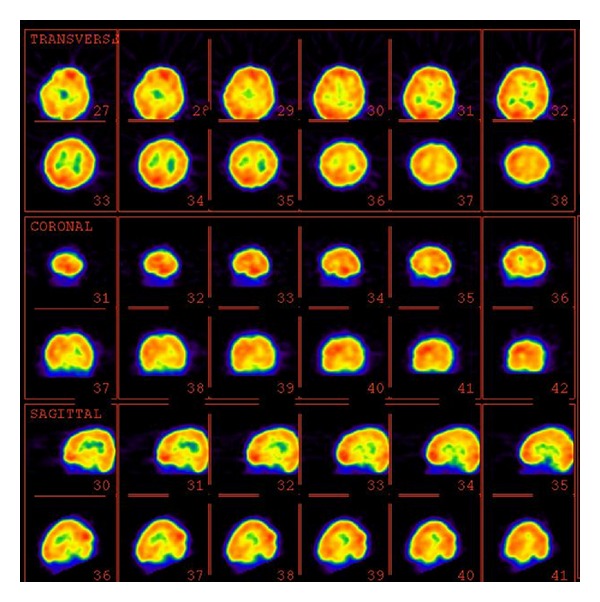
99mTc-ECD brain perfusion SPECT image of a 6-year-old boy with Landau-Kleffner syndrome (LKS). There is moderate hypoperfusion in the left temporal, left parietal, and left inferior frontal regions and also mild hypoperfusion in the right inferior parietal area. The upper rows indicate transverse 99mTc-ECD SPECT images. The middle rows indicate coronal 99mTc-ECD SPECT images. The lower rows indicate sagittal 99mTc-ECD SPECT images.

A six-year-old right-handed male patient presented with normal early development of gross and fine motor skills, with normal early language development up to year 4. However, he developed a progressive receptive and expressive language disorder. He had no history of epilepsy, with the exception of one episode of a generalized tonic-clonic convulsion following a viral illness. His neurological examination was normal; and an EEG showed a spike and slow waves in both the right and left central regions. The cranial MRI was normal. A brain perfusion SPECT during the sleep state showed moderate decreased perfusion in the left temporal, left parietal, and left inferior frontal regions and mild hypoperfusion in the right inferior parietal area (Figure 2).

## 3. Discussion

Our study showed moderate hypoperfusion of the left frontotemporal region in both cases and right parietal hypoperfusion in the second case. Clinical epilepsy has no relationship to perfusion defect severity, because the second patient, who had only one episode of convulsion during his lifetime, had more perfusion defects than the first patient. However, a relationship seems to exist between aphasia and hypoperfusion, particularly in the second case. Cortical mapping following electrical stimulation has shown that there are three language areas in the dominant hemisphere: the posterior portion of the inferior frontal gyrus (Broca's area), the superior temporal gyrus and supramarginal gyrus (Wernicke's area), and the basal temporal language area in the fusiform gyrus. Language expression occurs via two regions of Broca's area and Wernicke's area, although comprehension in Wernicke's area is considered to be the main area in language reception. Typically in LKS, there is a severe disruption in both receptive and expressive speech, without evidence of motor dyspraxia, indicating that Wernicke's area is the region most likely to be affected [4].

Functional studies, like perfusion and electrophysiological studies, are prefererable to MRIs to investigate CNS function in different areas. Conventional structural CT and MRI studies have not revealed abnormal findings in LKS [2], except in the bilateral volume reduction of the superior temporal areas (26 to 51%), where Wernicke's area is localized [1].

The EEG is considered to be a basic component in the diagnosis of LKS, and it has always shown abnormal epileptic discharges in children with LKS; however, clinical seizures are present in only 70% of the patients. EEG spikes and spike-wave discharges are widespread or multifocal or with shifting predominance but are mostly temporal (in 85% of cases) and unilateral (in 15%), which usually exacerbated during sleep, occasionally with a continuous pattern [5]. EEG spectral and topographic mapping investigations have shown the high spectral powers of the delta, theta, and alpha waves over the fronto-centro-parietal region. These characteristics have been applied to suggest electrophysiological dysfunction of the fronto-centro-parietal regions as a major pathogenic feature of Landau-Kleffner syndrome (LKS) [2]. What is more, the likely pathophysiological relations between epilepsy, cognition, sleep macro- and microstructure, and sleep disorders by Parisi et al. were performed which demonstrated that improvement in the long-term cognitive-behavioural prognosis of children with epilepsy needs both good sleep quality and good seizure control [6].

Metabolic and perfusion imaging have produced various results with respect to the type and time of the study (like SPECT versus PET), sleep versus awake state, and ictal activity [5]. O'Tuama et al. evaluated perfusion abnormalities in 5 children with LKS, and they showed a similar pattern of hypoperfusion in 4 patients with predominantly involved right or left temporoparietal lobes. In all cases, the temporal cortex was primarily involved, although the abnormality extended into the adjacent part of the parietal lobe. Within the temporal lobes, the abnormality was most evident in the superior and lateral aspects and predominated in the region of the Sylvian fissure. Patient 2 showed two separate areas of abnormal perfusion asymmetry involving the inferior and posterosuperior aspects of the left and right parietal cortex, respectively [2].

SPECT scans of children with LKS showed hypoperfusion in the right and/or left temporoparietal areas during the waking state [4]. This pattern is similar to that in children with congenital dysplasia, without an epileptiform discharge in the EEG [4]. O'Tuama et al. reported hypoperfusion in Broca's area of the frontal lobe in patients with congenital dysplasia [2]. This finding suggests that the perisylvian cortex is the main structure which is involved in the pathogenesis of LKS [2].

However, Harbord et al. showed two different perfusion patterns in 5 patients with LKS. Hyperperfusion of the left temporoparietal region (Wernicke's area) was seen in three patients with a history of clinical seizures that underwent SPECT scans at the time of the seizure or when electrophysiological seizure activity was very frequent. The findings suggest that the area of hyperperfusion originated from the site of the seizure activity. However, hypoperfusion of the right or left temporal and/or parietal lobes was reported in awake patients, without a history of any clinical seizures [4].

Rintahaka et al., using a PET scan, reported moderate hypometabolism in the right and left thalamus and frontal and temporal cortices, mild hypometabolism in the parietal and anterior cingulate cortices, and severe hypometabolism in the bilateral occipital region. In a repeat PET examination done during sleep, in which continuous spike-waves during slow-wave sleep were seen, the only dissimilarity observed compared to the awake study was a marked bilateral increase in temporal cortex metabolism. The awake interictal PET in the second child was normal, except for mildly increased relative glucose metabolism in the left inferior temporal region. The sleep PET study with continuous spike-waves during slow-wave sleep in this child revealed hypermetabolism in both temporal regions; however, this was more pronounced, with a wider distribution, in the left temporal cortex [7].

These findings suggest that epilepsy originating from the temporal lobe showed increased signals in SPECT and PET. However, a limited study evaluated brain perfusion during epilepsy by SPECT or PET, which may lower the chance of the detection of hyperperfusion areas other than temporal that contribute to epilepsy, because the EEG showed a generalized distribution of epileptiform discharge, which commonly occurs in LKS. Morover, simultaneous SPECT-EEG recording in our study was impossible which may have influenced the results.

As mentioned above, the hypoperfusion area mainly occurs in the speech areas of the brain, but it does not* necessarily* occur in a recognized speech area of the brain and may not indicate the site of the abnormality causing LKS [4]. Discussion about the correlation or incoherence of aphasia and epilepsy with an abnormal perfusion area in is not lacking. Holmes et al. believed that abnormal cerebral perfusion in the temporal lobe independently caused both seizures and language disorders in LKS [8]. Rintahaka et al., by PET scan, showed that LKS is a generalized cerebral disease with a focal lesion that predominantly involves the perisylvian area [7], although they did address the generalized histological examination of patients affected by LKS. It is probable that regions other than those related to speech, like the occipital region, may contribute to epilepsy.

In this report, a discrepancy between affected regions compared to the areas expected based on the clinical symptoms is observed. It may be due to this concept that nuclear medicine modalities can easily distinguish a process because it is based on functional processes which are morphologically indistinguishable and even may not be obvious clinically [9–12].

Another factor that may complicate this correlation is the lateralization of speech areas following speech area lesions [13]. After an injury to the speech area, another area of the brain compensates for the damaged area [13]. The fact that this phenomenon is not, or slightly, seen in LKS can indicate that cerebral pathology in LKS may be more of a diffuse process than a limited pathology of the brain, although many authors believe that LKS is a focal brain lesion. The theory of generalized cerebral disease could explain the limited improvement in aphasia in LKS.

Overall, asymmetrical temporoparietal perfusion in the brain SPECT is a main finding of LKS and may be a helpful feature in differentiating from childhood epileptic and/or aphasic syndromes. However, the brain SPECT is not a useful method to determine the basic pathogenesis of this syndrome.
